# Diseases of the Oral Mucous Membrane

**Published:** 1892-04

**Authors:** J. D. Patterson

**Affiliations:** Kansas City, Mo.


ARTICLE IV.

### DISEASES OF THE ORAL MUCOUS MEMBRANE.

BY J. D. PATTERSON, D. D. S., KANSAS CITY, MO.

Read before the American Dental Association, 1891.

This subject has been very much neglected, because its importance has been underrated. Until the agitation brought about by investigation of the disease called pyorrhœa alveolaris there had been little attention paid to diseases of the mucous membrane. Since then investigators have recognized more the importance to the dental practitioner of a thorough knowledge of the etiology and pathology of these complaints.

The inception of the irritation by which we have diseases of the oral mucous membrane may be divided as follows: Accidental, constitutional and hereditary.

I desire in this article to specially call your attention to some of the accidental causes. They are very numerous. The irritation may be mechanical alone, or it may be from the invasion of micro-organisms, from absence of hygienic care, from disease, from infection, or from functional disturbance without mechanical aid.

We will first consider the cause of disease by an arrest of its function. The dental profession has not given thought to this line of investigation so much as to the search for constitutional conditions, and therefore, I believe, have signally failed to explain the etiology of certain symptoms, especially in the early stages of pyorrhœa.

In glancing at the anatomy of the membrane, it may be described as follows: A layer of epithelium; a connective tissue frame-work and glands underneath, separating the epithelial layer and provided with blood-vessels, lymphatics, and nerves. Whatever interferes with and changes the normal condition of the membrane may overcome its safeguards, and disease is the result.

Any marked deviation from the normal heat of the tissues—namely, 98°—results in morbid changes. Heat-production goes on all over the body. When any of the tissues are subject to cold, the production is stopped, and morbid action may occur. In the mouth the contraction of the membrane is the first step, accompanied by stoppage of the mucous secretion. Then we have increased nutrition, an over-production of the mucus secretion, a slightly reddened tissue from the same cause, an abundant effusion of serum escapes. As the inflammation progresses, we see the discharge becoming a purulent one. Micro-organisms now make their invasion, and assist in the advancing stages of the disease. These soon extend below the epithelium and the membrane swells and thickens.

Let it be borne in mind that up to an advanced stage there is not, of necessity, any presence of pathogenic or non-pathogenic micro-organisms.

The hereditary condition of tissue and constitutional environments are in these cases merely predisposing factors. In some cases it is not to be doubted that constitutional conditions figure largely, but therc are greater factors which explain other symptoms without seeking in the field of bodily conditions.

I pass now to another irritant—the prevalent practice of mouth-breathing. Leaving out of the question the change in the temperature, we find that the air inspired rapidly dries the surface of the mucous membrane, stopping the mucus-ducts until the same train of symptoms from destroyed functions already described is established. When a subject is presented who, from one cause or another, breathes through the mouth, the operator may at once predict the condition of the mucous membrane. Here we have again a numerous class of cases where undeniably the disease has arisen from the gradual drying of the naturally moist lubricating surface by the introduction of an atmosphere whice is not normal to the tissue.

The condition that is described as arising from the interference with the functions is not a result of mouth-breathing alone. It may result from different causes. It is seen in fiction from catarrhal, croupous, diphtheritic, or tubercular surroundings. It is superinduced sometimes by medicinal treatment, by mechanical irritants, and by uncleanliness. We find that while the cause of the described conditions may be varied, and that the cause will affect the malignancy of the disease, yet the symptoms are very similar.

This condition is a disturbed function. The chief symptom is an abnormal secretion of mucus.

In the oral cavity the train of symptoms we have described meets with anatomical surroundings which rapidly further the inflammatory process. We refer to the dental organs. These symptoms continue, and finally develop into what is called pyorrhœa alveolaris, which is a condition naturally resulting.

In diseases of the membrane of the mouth, resulting in what is familiarly known as pyorrhœa alveolaris, different authors are opposed to the term "catarrhal" in describing the clinical appearance. They consider catarrh a special disease of certain membranes, which ultimately leads to ulceration. It is not so at all. It is simply a condition of discharge, and not a disease of special cause.

Another somewhat prevalent idea entertained by the profession is that there is a peculiar systemic condition under which the patient becomes liable to catarrhal inflammation.

Dr. Bosworth says, "I know of no such condition, and there is no good ground for the assertion. My clinical observation also fails to justify this view in any manner." ("Diseases of the Throat and Nose," p. 101.)

Another argument against classifying these diseases of the oral mucous membrane as "catarrhal" comes from those who lay great stress upon climate influence. They find in climates where catarrh is almost unknown, many cases of pyorrhœa, and, on the contrary, where catarrhs are endemic, find no increase in the number of those suffering from discharge from the oral mucous membrane.

I think this statement should be considered a very loose one. It was formerly supposed that more of these diseases were in America than in Europe, but this has been disproved. There can be no question that the change of climate works wonders in catarrhal trouble, but that climate influence is an important factor in its causation is open to question.

In leaving the question for your discussion, I leave with you the following considerations:

1st. That the inception of disease in the membrane, where the cause is obscure, is often to be found in the arrest of function in that membrane by shock, by catching cold, or by mouth breathing, by which the moist lubricated membrane is destroyed, rather than by hereditary conditions, or the invasion of micro-organisms.

2nd. That the irritated mucous membrane, which we find in abnormal quantities, is not pathognomonic of any special irritation, but is simply a condition.

3rd. That this condition is properly termed catarrh.

4th. Catarrh is not a special disease; merely a condition of disease.

5th. That the name pyorrhœa alveolaris simply denotes a stage of this catarrh in the oral mucous membrane, and may result from a great variety of causes.—*Dental Cosmos.*

